# Info-gap theory to determine cost-effective eradication of invasive species

**DOI:** 10.1038/s41598-023-29571-3

**Published:** 2023-02-16

**Authors:** Yang Liu, Melissa L. Thomas, Grey T. Coupland, Penghao Wang, Dan Zheng, Simon J. McKirdy

**Affiliations:** 1 Harry Butler Institute, Murdoch University, Perth, WA 6150 Australia; 2 Qingdao Agricultural University, Shandong, 266109 People’s Republic of China; 3 Food Futures Institute, Murdoch University, Perth, WA 6150 Australia

**Keywords:** Ecological modelling, Invasive species

## Abstract

Invasive species eradication campaigns often fail due to stochastic arrival events, unpredictable detectability and incorrect resource allocation. Severe uncertainty in model parameter estimates may skew the eradication policy results. Using info-gap decision theory, this research aims to provide managers with a method to quantify their confidence in realizing successful eradication of particular invasive species within their specified eradication budgets (i.e. allowed eradication cost) in face of information-gaps. The potential introduction of the Asian house gecko *Hemidactylus frenatus* to Barrow Island, Australia is used as a case study to illustrate the model. Results of this research demonstrate that, more robustness to uncertainty in the model parameters can be earnt by (1) increasing the allowed eradication cost (2) investment in pre-border quarantine and border inspection (i.e. prevention) or (3) investment in post-border detection surveillance. The combination of a post-border spatial dispersal model and info-gap decision theory demonstrates a novel and spatially efficient method for managers to evaluate the robustness of eradication policies for incursion of invasive species with unexpected behaviour. These methods can be used to provide insight into the success of management goals, in particular the eradication of invasive species on islands or in broader mainland areas. These insights will assist in avoiding eradication failure and wasteful budget allocation and labour investment.

## Introduction

Ecological systems are under serious threat from invasive alien species’ (IAS) (also known as invasive species) through increasing international trade and reductions in trade barriers^1^. For oceanic islands, where there are no predators and endemics have not evolved anti-predator defence mechanisms, invasive species can cause significant harm^2^. Eradication of invasive species is the most suitable course of action, as only through the complete removal of the invasive species are island ecosystems able to fully recover^3^. Unfortunately, the amount of resources allocated to an eradication activity can influence whether the eradication program is successful^4^. Knowledge of the budget therefore could assist in planning eradications^5^.

In the Era of Globalization, aviation and shipping ports are regarded as major entry points for global trade and passenger transport^6^. Taxonomic and geographic distribution of introduced invasive species is highly dependent on trade and passenger routes^7^. Ignoring the interaction of spatially connected processes (e.g. pathways, regions and control policies), conservation results may significantly and stochastically deviate from the optimal control strategies^8^. Increasing research has focused on spatial IAS management policies (e.g.^8–10^), and have found that spatial heterogeneity and network linkage are necessary to trigger efficient spatial policies^11^.

Key determinants of eradication cost are size and distribution of the targeted population. Early detection could enable the invasive species to be detected and eradicated early when it is still a small population, but requires intensive post-border surveillance (surveillance, hereafter)^12^. Post-border surveillance refers to surveillance that is completed following final quarantine clearance on the island. Uncertainty in initial population size (and distribution) could potentially cause delays in management actions. In most studies of detection strategies, the size of the population to be eradicated is assumed as known^13^, with only a few researchers recognising that there is uncertainty relating to this variable (e.g.^14^). Mehta et al.^14^ incorporate the uncertainty in initial population size using sensitivity analysis with the assumption that probability distribution is known. However, initial population size is characterised by large knowledge-deficiencies that cannot be measured probabilistically. It is important for deep uncertainties in population size and distribution to be incorporated into decision making in order to quantify outcome performance and evaluate the robustness of the designed eradication program. For situations facing deep uncertainty, info-gap decision theory (IGDT) has proven to be useful^15,16^. This is the first application of IGDT to generate cost-effective, but also robust eradication policy to manage insular invasive species with limited budgets.

Info-gap decision theory^17^ is designed for Knightian uncertainty^18^, i.e. situations where probability distributions for future cases are not accessible, inappropriate or unreliable or with uncertain outcomes^19^. Thus, IGDT is also known as a non-probabilistic theory. Traditionally, probability mathematically represents either degree of belief (e.g. probability distribution of Bayesian statistics) or likelihood (frequency) of events, based on which outcomes rank in various principles, e.g. maximum expected utility, minimum mean-square error^20^. While under Knightian uncertainty eluding distribution specification, Wald’s maximin is a common method to deal with uncertainty by minimizing worst outcome. Alternatively, IGDT offers a method to quantify the confidence in realising specified aspirations and enable the balance between them^21^.

Asian house gecko *Hemidactylus frenatus* Duméril & Bibron, 1836 (AHG) is native to South and South-East Asia. It has spread widely since the 1950s^22,23^ and is now considered one of the most widespread reptiles worldwide^24^. As this species is primarily spread via human mediated transport, *H. frenatus* typically spreads along a complex ‘spot-fire’ pattern, instead of a single invasion front^25^. In some areas, this species is known to displace native gecko species^26^, and has the potential to disrupt electrical equipment when the equipment is used as a source of thermoregulation by the gecko^27^.

**Figure 1:**
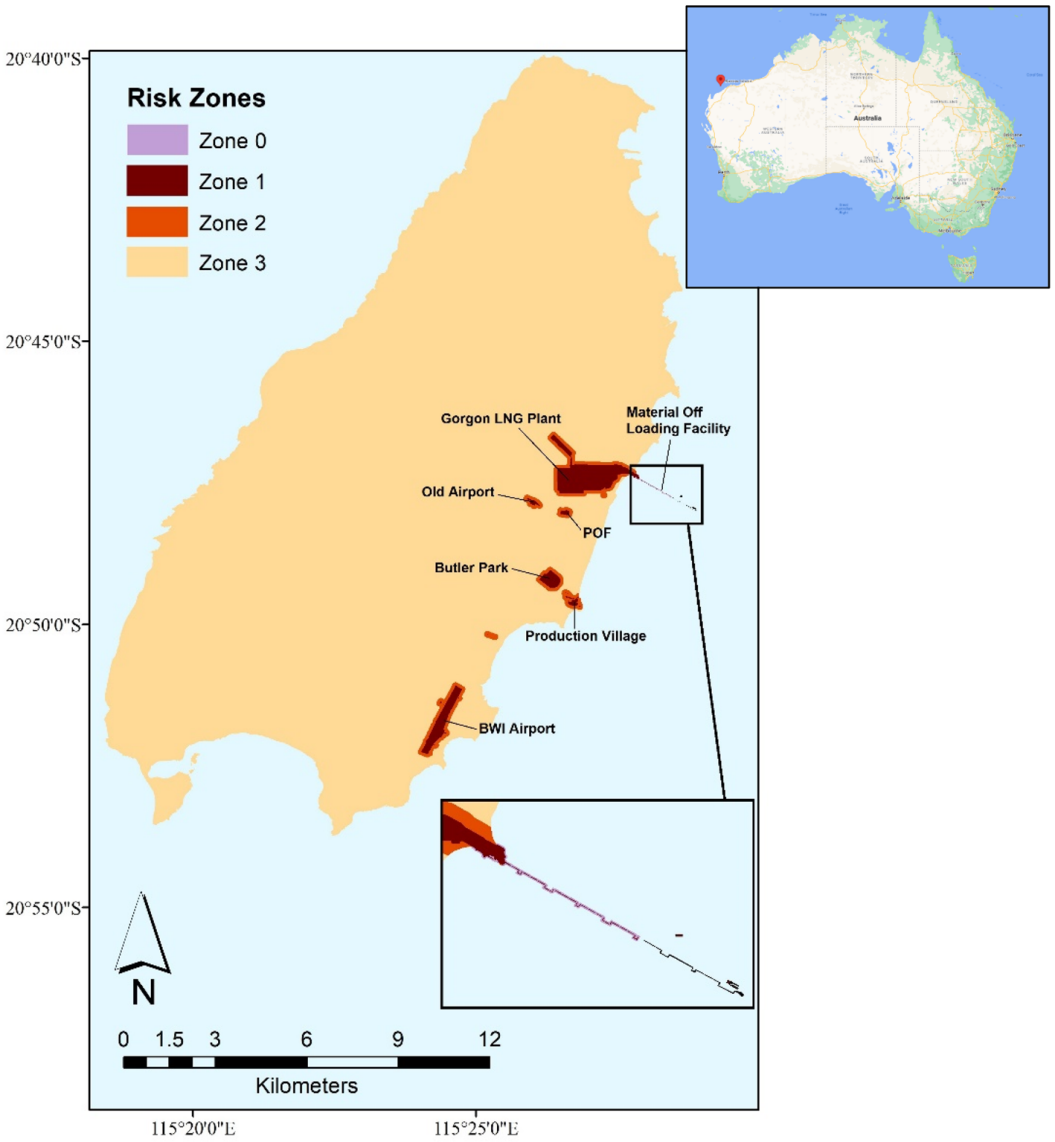
Quarantine invasion risk map of the Asian house gecko (AHG) on Barrow Island^12^. Inset map at the top right is the map of Australia with the location of Barrow Island shown as a red marker (https://www.google.com/maps). Zone 0 is the buffer area at Material Offloading Facility (MOF) (i.e. X-Blocs area). Zone 1 is the area with the highest occupancy probability, where the majority of the surveillance budget should be spent. Zone 2 is the secondary introduction area (100 m buffer area around Zone 1) and is considered the lower risk boundary for a species dispersing out of Zone 1. Zone 3 is the remaining island area where the AHG is less likely to establish prior to detection thus with no SSCs allocated.

Barrow Island (BWI) is located off the northwest coast of the Australian mainland (Fig. 1). It was declared a ‘Class A’ nature reserve in 1910 and is home to a rich diversity of species with high conservation value. Industrial development on the island has increased the risk of IAS incursion^28^. AHGs were first detected on BWI in 2015, with seven individuals found and successfully eradicated^12^. Eradicating AHG during the early stage of incursion is important, considering the low probability of eradication success and limitations of detection^29^. Also, the impacts this species has on the ecosystem and/or industry operations remains unclear. Eradication of AHG after it is established (i.e. ‘persisting long enough in the new region to be able to reproduce’^30^) could be costly and with no guarantee of success^31^.

Given the often-considerable financial investment in IAS eradication programs, it is important that programs are undertaken with confidence in their capacity to avoid possible long-lasting investment in permanent control or containment activities after eradication failure. The aim of this research is to apply IGDT to quantify confidence in successful eradication within specified eradication budgets (i.e. allowed eradication cost, hereafter) and provide a balance between confidence and allowed eradication cost. This research also provides insights into pre-border quarantine and border inspection (prevention, hereafter) and post-border surveillance to help raise confidence in eradication programs. Border inspection refers to the inspection of cargo and vessels etc. for any biosecurity risk materials at the border. The potential incursion of the invasive AHG on BWI, Western Australia is used to illustrate the model in this research. The AHG is one of the highest risk species for incursion onto BWI based on the number of interceptions of this species during pre-border quarantine and border inspection.

## Materials and methods

**Figure 2:**
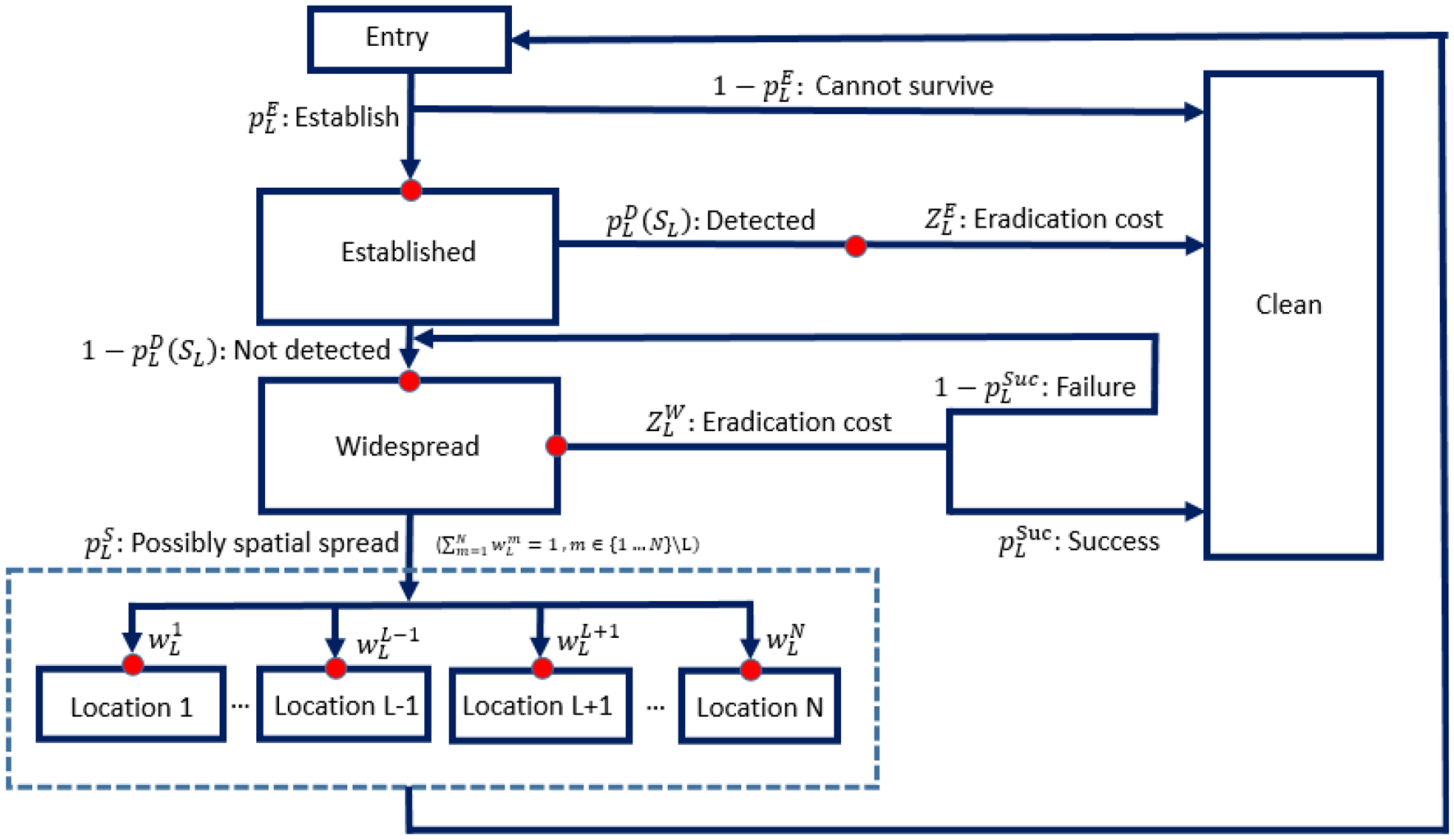
Post-border spatial dispersal modelling^10^. After an individual invasive species enters location $L$, the probability of its survival and establishment at location $L$ is $p_{L}^{E}$. The cost of non-survival is zero. The probability of it being detected after establishment with surveillance cost $S_{L}$ spent at location $L$ is $p_{L}^{D} (S_{L} )$. Once detected, it will be eradicated with eradication cost $Z_{L}^{E}$; if not, it will become widespread at location $L$, or even move to other potential locations $m = 1,2, \ldots ,n\backslash L$ with probability $p_{L}^{S} w_{L}^{m}$ ($p_{L}^{S}$ is a common coefficient that is specific to location $L$, $w_{L}^{m}$ is a spatial connection weight between location $L$ and $m$) and another loop process starts. It is assumed that when the invasive species becomes widespread at location $L$, the invasive species could be detected with 100% probability. The following eradication conducted with budget $Z_{L}^{W}$ (much higher than $Z_{L}^{E}$), may fail with probability $1 - p_{L}^{Suc}$. It should be noticed that $\sum\nolimits_{m = 1}^{N} {w_{L}^{m} = 1,} \, m \in \{ 1,2, \ldots ,n\} \backslash L$.

Recently, Long et al.^10^ outlined a post-border spatial dispersal model (Fig. 2) to optimize surveillance spatial allocation to minimize expected total management cost of four invasive species, whose spread on BWI could be promoted through human-mediated transportation. Their model is extended in this research by considering the Knightian uncertainty in the model parameter estimates and evaluating the robustness of various management policies to the Knightian uncertainty.

### Info-gap theory framework

Info-gap decision theory requires three components: (1) a system model used to represent the system consisting elements considered as the most significant; (2) a performance requirement which is actually a certain critical threshold made by decision makers; and (3) an uncertainty model to describe what is unknown about the system model parameters^17^.

The post-border spatial dispersal model (Fig. 2) has been used as the system model in this research. The eradication cost $C(EXP_{L} )$ of one individual IAS entering location $L$ is derived as Eq. (1), which is the weighted sum of the cost of survival and non-survival; the cost of non-survival is zero and that of survival and establishment $C(E_{L} )$ consists of the weighted sum of the cost of detection and the cost of non-detection at location $L$ (Eq. (2)). This research did not discount the eradication cost, assuming the eradication will be completed over a limited time on islands of suitable size.

1 $$C(EXP_{L} ) = P_{L}^{E} \times C(E_{L} )$$

where, $P_{L}^{E}$ is the probability of survival and establishment of an IAS at location $L$ and $P_{L}^{E} = \sum\nolimits_{Z} {(\frac{{A_{L,Z} }}{{A_{L} }}} P_{L,Z}^{E} )$ (where, $Z$ is the risk zone; $A_{L,Z}$ is the searching area of zone $Z$ of location $L$; $A_{L}$ is the searching area of location $L$; $P_{L,Z}^{E}$ is the survival probability of an IAS in zone $Z$ of location $L$); $C(E_{L} )$ is the cost of an IAS establishing at location $L$, defined as Eq. (2).

2 $$C\left( {E_{L} } \right) = P_{L}^{D} \left( {S_{L} } \right) \times C_{L}^{{{\text{nw}}}} + \left( {1 - P_{L}^{D} \left( {S_{L} } \right)} \right)\left( {\frac{1}{{P_{L}^{{{\text{Suc}}}} }} \times C_{L}^{w} + \frac{1}{{P_{L}^{{{\text{Suc}}}} }} \times \sum\limits_{{m \in \left\{ {1 \ldots {\text{n}}} \right\}\backslash {\text{L}}}} {P_{L}^{S} w_{L}^{m} C} \left( {E_{m} } \right)} \right)$$

where, $P_{L}^{D} (S_{L} )$ is the probability of an IAS detected given it is present in the surveillance area with surveillance cost $S_{L}$ used at location $L$; $C_{L}^{nw}$ is the eradication cost when the IAS is still localised (i.e. not widespread) at location $L$; $P_{L}^{Suc}$ is the probability of successful eradication of an IAS at location $L$; $C_{L}^{w}$ is the eradication cost when the IAS becomes widespread at location $L$; $p_{L}^{S}$ is a common coefficient of possibly spatial dispersal and specific to location $L$; $w_{L}^{m}$ is a spatial connection weight assigned to the destination location $m$ from location $L$; $C(E_{m} )$ is the cost of an IAS establishing at location $m$.

The detection probability $P_{L}^{D} (S_{L} )$ with surveillance cost $S_{L}$ used at each location is defined as Eq. (3) assuming that the distribution of both search effort and position of IAS is random according to search theory^32^. That is,

3 $$P_{L}^{D} (S_{L} ) = 1 - \sum\nolimits_{Z} {\frac{{A_{L,Z} }}{{A_{L} }}e^{{ - \sum\nolimits_{i} {K_{L} \frac{{F_{L,Z}^{i} }}{{A_{L,Z} }}\sigma_{L,Z}^{i} } \frac{{S_{L,Z}^{i} }}{{C_{L,Z}^{i} }}}} }$$

where, $i$ is the type of Surveillance System Component (SSC) used for surveillance detection; $K_{L}$ is the population threshold for detection at location $L$; $F_{L,Z}^{i}$ is the footprint (i.e. detection area covered by one unit of SSC deployment) of one unit of SSC $i$ in zone $Z$ of location $L$ ; $\sigma_{L,Z}^{i}$ is the detectability of SSC $i$ in zone $Z$ of location $L$, given that the invasive species is present in the SSC footprint; $C_{L,Z}^{i}$ is the cost per unit SSC $i$ in zone $Z$ of location $L$; $S_{L,Z}^{i}$ is the surveillance cost of SSC $i$ in zone $Z$ of location $L$.

The eradication cost at all potential invasion locations is then summarized as Eq. (4).

4 $$r = En \times \sum\nolimits_{L} {(\gamma_{L} \times C(EXP_{L} ))}$$

where, $En$ is the annual number of entries of IAS individuals of a particular species after border inspection to any of the locations (i.e. entry individuals hereafter); $\gamma_{L}$ is the probability of an IAS entry to location $L$ and $\sum\nolimits_{L} {\gamma_{L} } = 1$.

The system model results applied to calculate the eradication cost $r$ is then evaluated against a certain threshold value $r_{\alpha }$, requiring the eradication cost no more than $r_{\alpha } (r \le r_{\alpha } )$. $r_{\alpha }$ can be interpreted as a maximum budget, i.e. allowed eradication cost, that managers would like to allocate to an eradication activity at which the eradication activity is considered to always succeed over the horizon of uncertainty $\alpha$.

In this research, 43 uncertain parameters are considered, including $En$ (entry individuals of AHG), $\gamma_{L}$ (entry probability), $P_{L}^{D}$ (detection probability), $p_{L}^{S}$ (spatial dispersal probability), $P_{L}^{Suc}$ (probability of eradication success) at location $L(L = 1, \ldots ,6)$, and survival probability $P_{L,Z}^{E}$ in zone $Z = 0,1,2$ at location $L(L = 1, \ldots ,6)$ (see Fig. 1 and Supplementary Table S1 for zones and locations). 43-vectors $x = (x_{1} , \ldots ,x_{43} )$ are used to represent these uncertainty parameters and referred to $En,\gamma_{L} ,P_{L}^{D} , p_{L}^{S} ,P_{L}^{Suc} ,P_{L,Z}^{E}$ for simplicity. The nominal value (best estimates) of the parameters represented by vector $\tilde{x}$ , rely on the best available information that can be collected at present (see Supplementary Table S2 for SSC types and Supplementary Tables S3–S7 for nominal values and nominal input values of detection probability). The only available information about these parameter estimates is that they are all non-negative and all other parameters except $En$ have an upper boundary of one. In such a data-poor environment, it is difficult to determine the deviation of the best estimated value from their true value. Fractional-error uncertainty model $U(\alpha )$ describes a set of possible events within the horizon of uncertainty without considering the probability or frequency of outcomes^33^:

5 $$\begin{gathered} U(\alpha ) = \{ x:|\frac{{x_{n} - \tilde{x}_{n} }}{{\tilde{x}_{n} }}| \le \alpha ,x_{n} \ge 0,n = 1:43;\gamma_{L} \le 1,P_{L}^{D} \le 1,p_{L}^{S} \le 1,P_{L}^{Suc} \le 1,P_{L,Z}^{E} \le 1,L = 1:6,Z = 0:2\} ,\alpha \ge 0 \\ \\ \end{gathered}$$

assuming that the deviation is an unknown fraction $\alpha$ or less, i.e.

6 $$\tilde{x}_{n} - \tilde{x}_{n} \alpha \le x_{n} \le \tilde{x}_{n} + \tilde{x}_{n} \alpha$$

In this research, a set of nested uncertainty ranging from zero to one is applied given that the value of $\alpha$ is known.

### Robustness model

Following Ben-Haim^17^, robustness $\hat{\alpha }(r_{\alpha } ) = \max \{ \alpha :(\mathop {\max r}\limits_{{x_{n} \in U(\alpha )}} ) \le r_{\alpha } \}$ is defined as the largest info-gap at which eradication cost is ensured to be no more than $r_{\alpha }$. Let $m(\alpha )$ represents $\max r$ in the robustness function, as $m(\alpha )$ is the inverse function of $\hat{\alpha }(r_{\alpha } )$^12^, $m(\alpha )$ can be derived to plot the robustness curves. Function ‘fmincon’ in MATLAB R2018b^34^ was used to calculate the corresponding maximization with each value of $\alpha$.

The situations for conducting robustness analysis are organized as follows: $\tilde{K}_{L}$ at locations $L = 1,\ldots,6$ are assumed to be the same and set at 20, 8, 3 and $\tilde{E}n$ is set at 100, 50, 5, 1, to evaluate the robustness at various estimated population thresholds for detection and estimated entry individuals of AHG respectively. To evaluate the effects of surveillance budget on predicted eradication cost and corresponding robustness, the estimated surveillance cost is altered by (1) Optimal Spatial–optimal surveillance expenditure with spatial spread model applies when $\tilde{K}_{L} = 8$ (estimates from subject matter experts) and $\tilde{E}n = 5$ ^10^ (average number of individual AHG detected annually at the BWI border); (2) Doubling Optimal Spatial—doubling (200%) the number of SSCs deployed in (1) in each zone at each location; (3) Critical Spatial—minimising the number of SSCs in (1) in each zone at each location to achieve Gorgon Gas Development’s ministerial commitment (i.e. the surveillance program could detect an individual introduced species if it was present on the island due to activities of Gorgon Project (one of the world’s largest natural gas projects, operated by Chevron Australia), with a statistical power of detection of 0.8 or greater^35^); (4) Critical Local—using SSCs of surveillance program applied by Chevron based on local spread model^12^ to meet the ministerial requirement. This is to ensure the comparability of results of (3) and (4); and (5) No SSCs Spatial—allocating no SSCs using spatial spread model. The last scenario investigates the necessity of surveillance. The quantity of corresponding SSCs to be used in each situation is listed in Table 1.

**Table 1:** Quantity of corresponding Surveillance System Components (SSCs, see definitions of SSCs in Supplementary Table S2) to be used in each situation when estimated population threshold for detection is eight ($\tilde{K}_{L} = 8$) and annual number of entries of Asian house gecko individuals after border inspection to any of the locations on Barrow Island is five ($\tilde{E}n = 5$).

Situations	Unstructured surveys	Structured surveys	Gecko scat collections	EARS^a^ (non-networked)	Passive workers	EARS (networked)
(1) Optimal spatial	12	12	12	12	6	17,762
(2) Doubling optimal spatial	24	24	24	24	12	35,524
(3) Critical spatial	23	12	12	138	6	13,075
(4) Critical local	62	12	53	165	1000	324
(5) No SSCs spatial	0	0	0	0	0	0

‘Spatial’ indicates the situation with spatial spread model applies and ‘Local’ indicates the situation with local spread model applies. ‘Critical’ indicates that the surveillance programme is designed to critically achieve Gorgon’s ministerial commitment.

^a^EARS: Environmental Acoustic Recognition Sensors. These are devices that autonomously listen for the call of an Asian House Gecko and relay any detections to the end user through a user interface.

## Results

**Figure 3:**
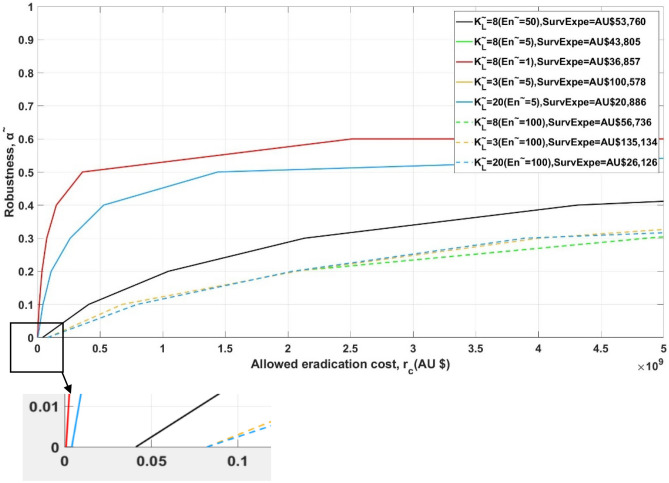
Robustness curves using the spatial dispersal model. $\tilde{K}_{L}$ is the estimated population threshold for detection, assumed to be the same at each location $L = 1, \ldots ,6$ and $\tilde{E}n$ is the estimated annual number of entries of Asian house gecko after border inspection to any of the locations on Barrow Island. The corresponding optimal surveillance cost in each situation are listed as ‘SurvExpe’ in the legend. The lines with the same value of $\tilde{E}n$ are aligned with each other.

**Figure 4:**
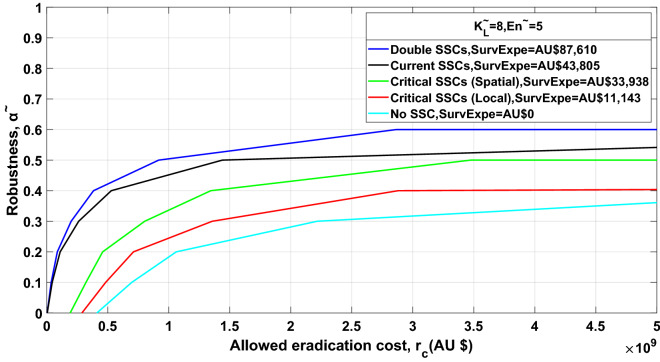
Robustness curves for situations shown in the legend. In each situation, the population threshold for detection of Asian house gecko at each location is estimated at 8 (i.e. $\tilde{K}_{L} = 8$ for $L = 1, \ldots ,6$) and the annual number of entries of AHG individuals after border inspection to any of the locations on Barrow Island is estimated at 5 ($\tilde{E}n = 5$). All lines, except for the red line (based on local spread model), are based on spatial spread model. The optimal allocation of Surveillance System Components (SSCs) in each zone at each location is denoted as ‘Optimal Spatial’. The application of double (200%) and no SSCs in each zone at each location is denoted as ‘Doubling Optimal Spatial’ and ‘No SSCs Spatial’ separately. The ‘Critical Spatial’ and ‘Critical Local’ are applied to meet Chevron’s ministerial requirements. The ‘SurvExpe’ indicates the corresponding estimated surveillance cost in each situation.

Robustness (y-axis, Figs. 3 and 4) shows the greatest parameter estimates error, up to which the estimated eradication cost is always lower than the allowed eradication cost required by the managers (x-axis, Figs. 3 and 4). For example, robustness of 0.10 (black line in Fig. 3) indicates that uncertainty parameter estimates error, up to ± 10%, will never jeopardize the corresponding allowed eradication cost, which is AU$410 million. The converging points of each robustness curve with horizontal axis (Figs. 3 and 4) present the predicted eradication cost with estimated parameter values and show no robustness to uncertainty (i.e. zeroing property). For example, the black line (Fig. 3) indicates an estimated eradication cost of AU$41 million, the robustness of which is zero. This predicted eradication cost is regarded as unreliable due to the severe uncertainty associated with the input parameters.

Immunity to underlying uncertainty (i.e. robustness) could be gained through increasing allowed eradication costs (Figs. 3 and 4), with a plateau at 0.6. That is, robustness of more than 0.6 could not be gained even if managers further increase allowed eradication cost. Managers may decide the allowed eradication cost to be allocated based on their preferred robustness to the underlying parameter uncertainties. Alternatively, managers could evaluate the robustness of their allowed eradication cost. This is called the trade-off property of IGDT.

In addition to gaining robustness through increasing allowed eradication costs, how much robustness could be gained is also reliant on the estimated entry individuals of AHG onto BWI (i.e. $$\tilde{E}n$$). As Fig. 3 indicates, when the estimated population threshold $$\tilde{K}_{L}$$ is constant, confidence in realizing successful eradication (i.e. robustness) increases when the estimated entry individuals $$\tilde{E}n$$ decreases. For example, an error of ± 51% ($$\tilde{E}n = 1,\tilde{K}_{L} = 8$$), can be tolerated in estimated uncertainty parameters with the actual eradication cost guaranteed to be no greater than AU$500 million. This is larger than that of ± 12% when $$\tilde{E}n = 50,\tilde{K}_{L} = 8$$. When $$\tilde{E}n$$ is constant, the estimated population threshold $$\tilde{K}_{L}$$ for surveillance detection has no influence on the robustness of eradication policy (Fig. 3).

A decrease in the number of SSCs deployed, results in a corresponding decrease in the robustness of specific allowed eradication cost (Fig. 4). Doubling the number of SSCs (blue line in Fig. 4) is the robust dominant strategy that should be preferred by managers. When no SSC is deployed (blue line in Fig. 4) there is a substantial increase in the allowed eradication cost required for eradication activities to gain the same level of robustness. This is because when fewer SSCs are deployed, AHG are detected only once they are well established and at high numbers, at which point they become difficult to eradicate. The spatial spread model appears to be more robust than the local spread models to critically achieve Gorgon’s ministerial commitment (Fig. 4). This is not surprising considering that invasion probabilities between locations connected by transportation can be influenced by each another.

## Discussion

Eradication is the preferred option when re-invasion is unlikely and the initial population size is small^36^. Although complete prevention of re-invasion is not achievable, a reduction of invasion probability can be realised through allocating appropriate resources to prevention^37^. This study demonstrates that reducing border pressure (i.e. the annual number of entries of IAS individuals of a particular species after border inspection to any of the locations), thereby decreasing $\tilde{E}n$, could increase the robustness of specific allowed eradication cost. However, this relationship is not proportional (Fig. 3). Prevention is therefore necessary but cannot be completely guaranteed. For managers who are risk averse, resources tend to be put towards post-border strategies (e.g. surveillance) to solve existing issues, instead of prevention where there is uncertainty relating to invasion probability^38^. This research demonstrates that such risk-averse attitudes are detrimental when entry frequency of invasive species is high (i.e. high border pressure).

Insufficient trap deployment is one reason for possible eradication failure^39^, particularly relating to the capture of the last individuals^40^. Eradication of small India mongoose from Amami-Oshima, Japan, failed due to the difficulty of capturing individuals at low density^41^. Invasive gecko species have a high establishment success associated with high propagule pressure^29^. In the Caribbean region, preventing the introduction of invasive geckos is considered a priority, given that this species may go undetected at low densities and therefore have a higher chance of establishment and lower possibility of eradication^29^. Rapid response to prevent population growth and spread, accurate delimitation of infested area, and resource allocated to eradication are determinants of eradication success^42^. This research shows that increasing allowed eradication cost, thus enables more traps to be deployed and advances the probability of eradication success, gains managers more immunity (robustness) to underlying errors in the parameter estimates.

Greater surveillance effort can decrease predicted eradication costs by ensuring populations are detected when they are low in numbers and before they have spread^43^. Results of this research indicate that an increase in the quantity of SSCs deployed predicts a lower eradication cost and enables more robustness (Fig. 4). When no SSCs are deployed post-border, estimated eradication cost is significantly higher and there is less robustness associated with specific allowed eradication cost. For small populations, more resources need to be spent on surveillance to improve the probability of detecting IAS early^14^. This can be seen in Fig. 3 where the optimal surveillance costs associated with $$\tilde{K}_{L} = 3,8,20 \, (\tilde{E}n = 5)$$ are AU$100,578, AU$43,805 and AU$20,886 separately.

Results of this research lend support to spatial management policies to be more efficient. Incorporating the chance of incursion from surrounding locations connected by human-mediated transports (spatial spread model) in an insular area, results in more optimal surveillance expenditure, an increase in robustness, but a decrease in predicted eradication costs (Fig. 4). Spatial IAS management policies are thus desired, especially across heterogeneous landscapes.

Robust-optimal solutions may differ when various components of the model are structured in the uncertainty model^44^. As shown in Supplementary Section S1, modelling uncertainty in additional fundamental parameters in the nominal model of detection probability (Eq. (3)) is illustrated as an example. For Supplementary Fig. S1, info-gap analysis generates some different results from Fig. 3. That is, increasing the estimated population threshold for surveillance detection gains managers more robustness when the estimated entry individuals ($\tilde{E}n$) is constant (Supplementary Fig. S1). Investment in prevention to decrease the estimated entry individuals ($\tilde{E}n$) enables more robustness to be gained (Supplementary Fig. S1). Future research will consider how much should be spent on prevention, with uncertainty in cost-effectiveness of prevention included. Robustness plateaus at 0.7 (Supplementary Fig. S1), in comparison with that of 0.6 in Fig. 3, indicating more errors in parameter estimates could be tolerated without jeopardizing the performance requirement. Supplementary Fig. S2 exhibits the same outcome as Fig. 4, and demonstrates that more robustness could be earnt through: (1) using greater estimated surveillance effort with more SSCs applied; (2) increasing allowed eradication cost; (3) using spatial spread model to make spatial management policies.

The model in this research, as a fundamental model, can be extended to take both temporal and spatial factors into consideration to better tackle uncertainty. For example, detection probability is assumed to be constant without considering temporal (e.g. diurnal or seasonal) factors in this research. The surveillance system designed in this research performs over one year considering that the detectability of AHG may vary during a year due to seasonal changes. While in other research, detectability is considered to change with population size^43^, site visit length^45^ and the date of detection^13^. Further research into the biology of gecko species (e.g. reproductive cycle, Allee threshold and life expectancy) could change the decisions managers make following a detection. Methods in this research can also be applied in other areas aiming for robust eradication of IAS in ecology, including islands, farm and forest management, even for aquatic invasions resulting from transportation of IAS via vessels.

Impacts (ecological, economic and societal impacts) caused by IAS and environmental damage during eradication should also be considered in invasion management policies^46^. Quantification would require extensive quantitative data of the potential impacts of the IAS, which is currently unknown in managing the AHG in certain introduced environments. Overall, investing in prevention will reduce border pressure, while investing in surveillance will enable early detection and increase the chance of a successful eradication. All these methods are beneficial for earning more robustness. Results of this research reinforce previous research that has shown that prevention, surveillance and eradication programmes should never be considered in isolation, particularly when allocating limited budgets (e.g.^47,48^). Ignoring deep uncertainty may skew the optimal policy results. There is a need for further research into the robust resource allocation across the continuum of activities to ensure cost-effective, but also robust biosecurity management.

## Supplementary Information

Supplementary Information.

## Data Availability

Data available via the Dryad Digital Repository 10.5061/dryad.b8gtht7h0.

## References

[1] Peterson AT, Vieglais DA (2001). Predicting species invasions using ecological niche modeling: New approaches from bioinformatics attack a pressing problem. Bioscience.

[2] Atkinson IAE (2001). Introduced mammals and models for restoration. Biol. Conserv..

[3] Parkes JP, Panetta FD, Clout MN, Williams PA (2009). Eradication of invasive species: progress and emerging issues in the 21st century. Invasive Species Management: A Handbook of Principles and Techniques.

[4] Baker CM, Hodgson JC, Tartaglia E, Clarke RH (2017). Modelling tropical fire ant (*Solenopsis geminata*) dynamics and detection to inform an eradication project. Biol. Invasions.

[5] Simberloff D (2003). How much information on population biology is needed to manage introduced species?. Conserv. Biol..

[6] Hulme PE (2009). Trade, transport and trouble: Managing invasive species pathways in an era of globalization. J. Appl. Ecol..

[7] Meyerson LA, Mooney HA (2007). Invasive alien species in an era of globalization. Front. Ecol. Environ..

[8] Sanchirico JN, Albers HJ, Fischer C, Coleman C (2010). Spatial Management of invasive species: Pathways and policy options. Environ. Resour. Econ..

[9] Caplat P, Hui C, Maxwell BD, Peltzer DA (2014). Cross-scale management strategies for optimal control of trees invading from source plantations. Biol. Invasions.

[10] Long, Y., Van der Merwe, J., Thomas, M. L., McKirdy, S. & Kompas, T. Biosecurity for valuable environmental island assets: Spatial post-border surveillance for early detection*. Ecol. Econ.* forthcoming (2022).

[11] Kroetz K, Sanchirico JN (2015). The bioeconomics of spatial-dynamic systems in natural resource management. Annu. Rev. Resour. Econ..

[12] Liu Y, Wang P, Thomas ML, Zheng D, McKirdy SJ (2021). Cost-effective surveillance of invasive species using info-gap theory. Sci. Rep..

[13] Homans F, Horie T (2011). Optimal detection strategies for an established invasive pest. Ecol. Econ..

[14] Mehta SV, Haight RG, Homans FR, Polasky S, Venette RC (2007). Optimal detection and control strategies for invasive species management. Ecol. Econ..

[15] Moffitt LJ, Stranlund JK, Osteen CD (2008). Robust detection protocols for uncertain introductions of invasive species. J. Environ. Manage..

[16] Yokomizo H, Possingham HP, Hulme PE, Grice AC, Buckley YM (2011). Cost-benefit analysis for intentional plant introductions under uncertainty. Biol. Invasions.

[17] Ben-Haim Y (2006). Info-gap Decision Theory: Decisions Under Severe Uncertainty.

[18] Knight FH (1921). Risk, Uncertainty, and Profit.

[19] Regan HM (2005). Robust decision-making under severe uncertainty for conservation management. Ecol. Appl..

[20] Ben-Haim Y (2004). Uncertainty, probability and information-gaps. Reliab. Eng. Syst. Saf..

[21] Ben-Haim Y, Demertzis M (2016). Decision making in times of Knightian uncertainty: An info-gap perspective. Economics.

[22] Lever C (2003). Naturalized Reptiles and Amphibians of the World.

[23] Wilson JRU, Dormontt EE, Prentis PJ, Lowe AJ, Richardson DM (2009). Something in the way you move: Dispersal pathways affect invasion success. Trends Ecol. Evol..

[24] Torres-Carvajal O (2015). On the origin of South American populations of the common house gecko (Gekkonidae: *Hemidactylus frenatus*). NeoBiota.

[25] Hoskin CJ (2011). The invasion and potential impact of the Asian House Gecko (*Hemidactylus frenatus*) in Australia. Austral Ecol..

[26] Barnett LK (2017). Understanding Range Expansion of Asian House Geckos (Hemidactylus frenatus) in Natural Environments.

[27] Norval G, Mao J-J (2015). An instance of a house gecko (*Hemidactylus frenatus* Schlegel, 1836) utilizing an electrical timer for thermoregulation. IRCF Reptil. Amphib..

[28] Greenslade P, Burbidge AA, Lynch AJJ (2013). Keeping Australias islands free of introduced rodents Barrow Island. Pac. Conserv. Biol..

[29] Perella CD, Behm JE (2020). Understanding the spread and impact of exotic geckos in the greater Caribbean region. Biodivers. Conserv..

[30] Davis MA, Simberloff D, RejmÁNek M (2011). Invasion biology. Encyclopedia of Biological Invasions.

[31] García-Díaz P, Ross JV, Vall-llosera M, Cassey P (2019). Low detectability of alien reptiles can lead to biosecurity management failure: A case study from Christmas Island (Australia). NeoBiota.

[32] Koopman, B. O. Search and Screening. *Operations Evaluation Group (OEG) Report.* (1946).

[33] Grasinger M, O'Malley D, Vesselinov V, Karra S (2016). Decision analysis for robust CO_2_ injection: Application of Bayesian-Information-Gap Decision Theory. Int. J. Greenh. Gas Control.

[34] MathWorks. *MATLAB R2018b*. (MathWorks, 2018).

[35] Commonwealth Government of Australia. *Approval—Gorgon Gas Development (EPBC Reference: 2008/4178)*. (2009).

[36] Kalaris T, Gordh G, McKirdy S (2014). The role of surveillance methods and technologies in plant biosecurity. The Handbook of Plant Biosecurity: Principles and Practices for the Identification, Containment and Control of Organisms that Threaten Agriculture and the Environment Globally.

[37] Sharma S, Mckirdy S, Macbeth F, Gordh G, McKirdy S (2014). The biosecurity continuum and trade: Tools for post-border biosecurity. The Handbook of Plant Biosecurity: Principles and Practices for the Identification, Containment and Control of Organisms that Threaten Agriculture and the Environment Globally.

[38] Epanchin-Niell RS (2017). Economics of invasive species policy and management. Biol. Invasions.

[39] Gregg H (2007). Invasive rodent eradication on islands. Conserv. Biol..

[40] Parkes, J. Feasibility plan to eradicate Common mynas (*Acridotheres tristis*) from Mangaia Island, Cook Islands. *Landcare Research Contract Report LC0506/184.* (2006).

[41] Barun A, Simberloff D, Simberloff D, RejmÁNek M (2011). Carnivores. Encyclopedia of Biological Invasions.

[42] Pluess T (2012). When are eradication campaigns successful? A test of common assumptions. Biol. Invasions.

[43] Epanchin-Niell RS, Haight RG, Berec L, Kean JM, Liebhold AM (2012). Optimal surveillance and eradication of invasive species in heterogeneous landscapes. Ecol. Lett..

[44] Rout TM, Thompson CJ, McCarthy MA (2009). Robust decisions for declaring eradication of invasive species. J. Appl. Ecol..

[45] Hauser CE, McCarthy MA (2009). Streamlining 'search and destroy': Cost-effective surveillance for invasive species management. Ecol. Lett..

[46] Epanchin-Niell RS, Hastings A (2010). Controlling established invaders: Integrating economics and spread dynamics to determine optimal management. Ecol. Lett..

[47] Moore JL (2010). Protecting islands from pest invasion: Optimal allocation of biosecurity resources between quarantine and surveillance. Biol. Conserv..

[48] Rout TM, Moore JL, Possingham HP, McCarthy MA (2011). Allocating biosecurity resources between preventing, detecting, and eradicating island invasions. Ecol. Econ..

