# Supplementary material for: Info-gap theory to determine cost-effective eradication of invasive species
Source: Sci Rep. 2023 Feb 16;13:2744. doi: 10.1038/s41598-023-29571-3 (PMC9935532; doi:10.1038/s41598-023-29571-3)
Supplement: Supplementary file 1 — Supplementary Information. [file 41598_2023_29571_MOESM1_ESM.pdf]

Info-gap theory to determine cost-effective eradication of invasive species

Yang Liu[1,2*], Melissa L. Thomas[1], Grey T. Coupland[1], Penghao Wang[1,3], Dan Zheng[2] and Simon J. McKirdy[1]

[1] *Harry Butler Institute, Murdoch University, Perth, WA 6150, Australia*

[2] *Qingdao Agricultural University, Shandong 266109, P.R. China*

[3] *Food Futures Institute, Murdoch University, Perth, WA 6150, Australia*

* *Corresponding author*

E-mail: Y.Liu@murdoch.edu.au

Supplementary Information

Table S1. Entry points for an Asian House Gecko incursion on Barrow Island, Western Australia. Data were provided by Chevron Australia

Points of entry	Description
MOF	Material Offloading Facility, where material is offloaded from vessels
Airport	BWI airport, where airplanes land and passengers transit to BWI accommodation
Old Airport	Old airplane landing strip that is now used for temporary laydown of cargo and part of which is a waste treatment plant
Accommodation	Two accommodation facilities on BWI; (1) Butler Park and (2) Production Village. These have been grouped together as a single entry site
POF	Permanent Operating Facility, which is the main operating offices for the gas treatment plant
Gorgon LNG Plant	Gorgon Liquefied Natural Gas Plant

Table S2. Definition of Surveillance System Components (SSCs). Data were provided by Chevron Australia

Surveillance System Components (SSCs)	Description
Non-networked Environmental Acoustic Recognition Sensors (EARS)	A device that can detect the multiple chirp calls of *Hemidactylus frenatus*. This device requires manual downloading of calls and timestamp of calls.<br>One SSC equals 1 EAR recording for 1 night.
Networked EARS	A networked device that can detect the multiple chirp calls of *Hemidactylus frenatus*. This device is powered by solar panels and provides notifications to an end user via a user interface when a suspect call is detected.<br>One SSC equals 1 EAR recording for 1 night.
Gecko scat collections	Search of a likely habitat area for signs of an introduced gecko. Areas searched include vertical surfaces near lights. DNA from scat samples is sequenced to determine species identity (see Thomas et al.[1]). Sequences are compared to taxa from the international sequence database (GenBank (ncbi.nlm.nih.gov/genbank/)), the Helix vertebrate database and the Barrow Island specific database for *Gekkonidae* and *Scincidae* species.

	One SSC equals a 100 m^2 area searched for gecko scats or a 1 hr long search.
Biologist structured surveys	A formal biological survey of an area looking for signs of non-indigenous vertebrate or invertebrate species. Signs may include tracks, scats, auditory calls, burrows, eye shine, eggs or individuals.<br>One SSC is approximately 5,000 m^2 area.
Biologist unstructured surveys	The biologist undertaking surveillance on Barrow Island may detect a potential *Hemidactylus frenatus* in his/her personal space when travelling around the island, but when not performing a biologist structured survey.
Passive workers [a]	Members of the island workforce who have not had any formal training to detect a *Hemidactylus frenatus* (aside from the induction), but may notice an unusual vertebrate or invertebrate in their personal work or recreation area and hand it into quarantine.<br>One SSC is equivalent to 1 personnel's work on site at any time on Barrow Island during 1 year.

[a] The maximum number of passive workers is 1,000. This number could vary depending on the activities being undertaken on the island.

Table S3. Sigma (σ), footprint and unit cost of various Surveillance System Components (SSCs) at different locations and zones on Barrow Island, Western Australia. Data were based on expert elicitation coordinated by Chevron Australia.

**Surveillance System Components (SSCs)**	**Sigma Z1** [a]	**Sigma Z2**	**Sigma Z0 ***	**Footprint** [b] **(m^2)**	**Cost** [c] **(AU$)**
EARS (non-networked)	0.4	0.4	0.2	300	5.5
EARS (networked)	0.45	0.45	0.45	300	2.3
Gecko scat collections	0.74	0.25	0.02	100	110
Biologist structured surveys	0.6	0.2	0.07	5000	110
Biologist unstructured surveys	0.3	0.01	0.035	3000	20
Passive workers	0.05	0	0.01	10	1.1

* Z0 only occurs at the Material Offloading Facility (MOF). [a] Sigma is the detection probability of SSCs given invasive species present in the footprint. [b] Footprint is the area in which an Asian house gecko can be detected with a single unit of SSC. [c] Cost is per unit of SSCs.

Table S4. Surveillance area of each location on Barrow Island, Western Australia. Data were provided by Chevron Australia

**Surface area (m^2)**	**Accommodation**	**POF**	**Airport**	**Old Airport**	**Gorgon LNG Plant**	**MOF**	**Total**
Z1[a]	250,308	60,735	5,011	7,959	55,713	19,826	399,552
Z2	422,191	90,812	72,701	136,467	698,178	0	1,420,348
Z0	0	0	0	0	0	300,000	300,000

[a] The surface area of Z1 does not refer to the entire Zone 1 area on the quarantine invasion risk map (Fig. 1), but only the specified habitat where Asian house gecko would be detected (i.e. building area).

Table S5. Incursion probability to each location on Barrow Island, Western Australia. Data were based on expert elicitation coordinated by Chevron Australia

**Incursion probability**	**Accommodation**	**POF**	**Airport**	**Old Airport**	**Gorgon LNG Plant**	**MOF**
	0.01	0.03	0.10	0.13	0.03	0.70

Table S6: Survival probability in each zone at each location on Barrow Island, Western Australia. Data were based on expert elicitation coordinated by Chevron Australia

**Survival probability**	**Accommodation**	**POF**	**Airport**	**Old Airport**	**Gorgon LNG Plant**	**MOF**
All Buildings	0.65	0.65	0.65	0.65	0.65	0.5
X-blocs	0	0	0	0	0	0.25
100m Buffer on Tenure	0.25	0.25	0.25	0.25	0.25	0.25

Table S7. Eradication cost at each location on Barrow Island, Western Australia. Data were provided by Chevron Australia

**Eradication cost (AU$1000)**	**Accommodation**	**POF**	**Airport**	**Old Airport**	**Gorgon LNG Plant**	**MOF**
1) with early detection	3,000	3,000	3,000	3,000	3,000	3,000
2) when widespread	150,000	150,000	150,000	150,000	150,000	150,000

Section S1: Additional fundamental uncertainty parameters

Here we model uncertainty using additional fundamental parameters of the nominal model of detection probability (Supplementary Eq. (S1)). The model of detection probability can also be found as Eq. (3) in the manuscript.

$$P_L^D(S_L) = 1 - \sum_Z \frac{A_{L,Z}}{A_L} e^{-\sum_i K_L \frac{F_{L,Z}^i}{A_{L,Z}} \sigma_{L,Z}^i \frac{S_{L,Z}^i}{C_{L,Z}^i}} \qquad \text{(S1)}$$

Where,

L is the potential locations of the AHG entering (Fig. 1, Supplementary Table S1);

Z is the risk zone ($Z = 0,1,2$) (Fig. 1);

i is the type of Surveillance System Component (SSC) for surveillance detection ($i = 1,2...,6$) (Supplementary Table S2);

$A_{L,Z}$ is the searching area of zone Z of location L;

A_L is the searching area of location L;

K_L is the population threshold for detection at location L;

$F_{L,Z}^i$ is the footprint of one unit of SSC i in zone Z of location L;

$\sigma_{L,Z}^i$ is the detectability of SSC i in zone Z of location L given the presence of invasive species in the SSC footprint;

$C_{L,Z}^i$ is the cost per unit SSC i in zone Z of location L;

$S_{L,Z}^i$ is surveillance cost of SSC i in zone Z of location L.

All the other uncertainty parameters, except for detection probability shown in Eq. (5) in the manuscript, are kept as uncertain. We now use 259-vectors $x = (x_1, ..., x_{265})$ to represent uncertainty parameters, containing En (entry individuals of AHG), γ_L (entry probability), p_L^S

(spatial spread probability), P_L^{Suc} (probability of eradication success) at location $L(L=1,...,6)$, and survival probability $P_{L,Z}^E$ in zone $Z=0,1,2$ at location $L(L=1,...,6)$ as in the manuscript, as well as K_L (population threshold) at each location L, $F_{L,Z}^i$ (footprint) and $\sigma_{L,Z}^i$ (detectability) of one unit of SSC i in zone Z of location L. But we refer to $En, \gamma_L, K_L, F_{L,Z}^i, \sigma_{L,Z}^i, P_L^{Suc}, p_L^S, P_{L,Z}^E$ for simplicity.

As in the manuscript, the fractional-error uncertainty model can be expressed as

$$U(\alpha)=\{x:\left|\frac{x_n-\tilde{x}_n}{\tilde{x}_n}\right|\le\alpha, x_n\ge 0, n=1:259; \gamma_L\le 1, \sigma_{L,Z}^i\le 1, p_L^S\le 1, P_L^{Suc}\le 1, P_{L,Z}^E\le 1,$$
$$i=1:6, L=1:6, Z=0:2\}, \alpha\ge 0$$

(S2)

Following are the robustness curves with the additional fundamental parameters included in various situations.

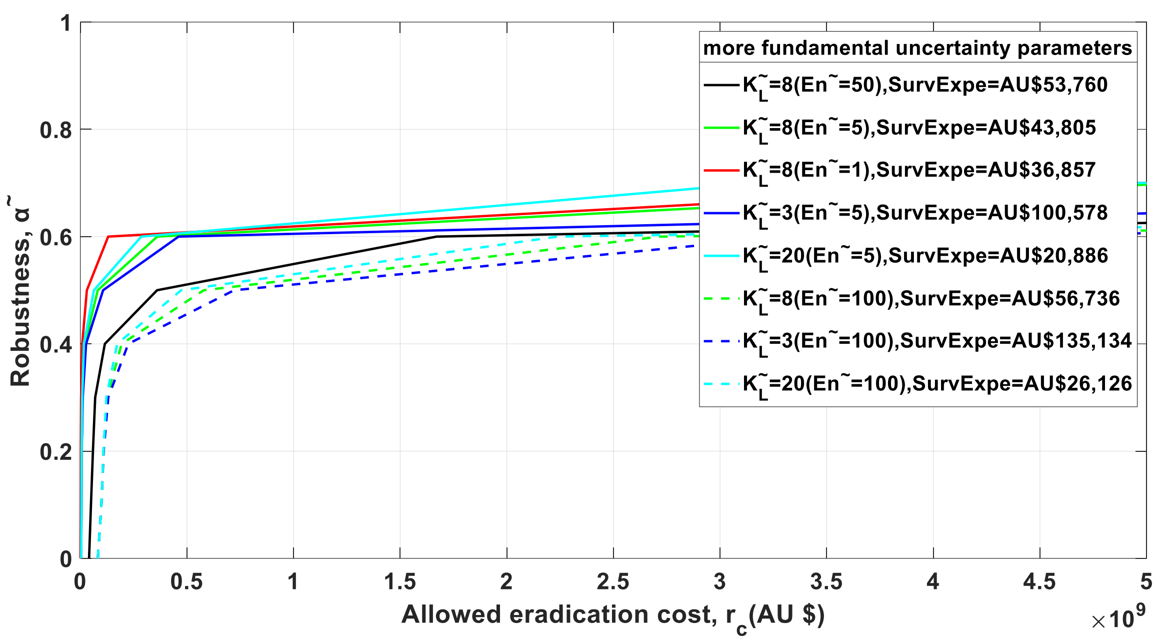

Figure S1. Robustness curves using spatial spread model with uncertainties modelled using additional fundamental parameters. $\tilde{K}_L$ is the estimated population threshold assumed to be the same at each location $L = 1,...,6$ and $\tilde{E}n$ is the estimated annual number of entries of Asian house gecko individuals after border inspection to any of the locations on Barrow Island. The corresponding optimal surveillance cost in each situation is listed as 'SurvExpe' in the legend

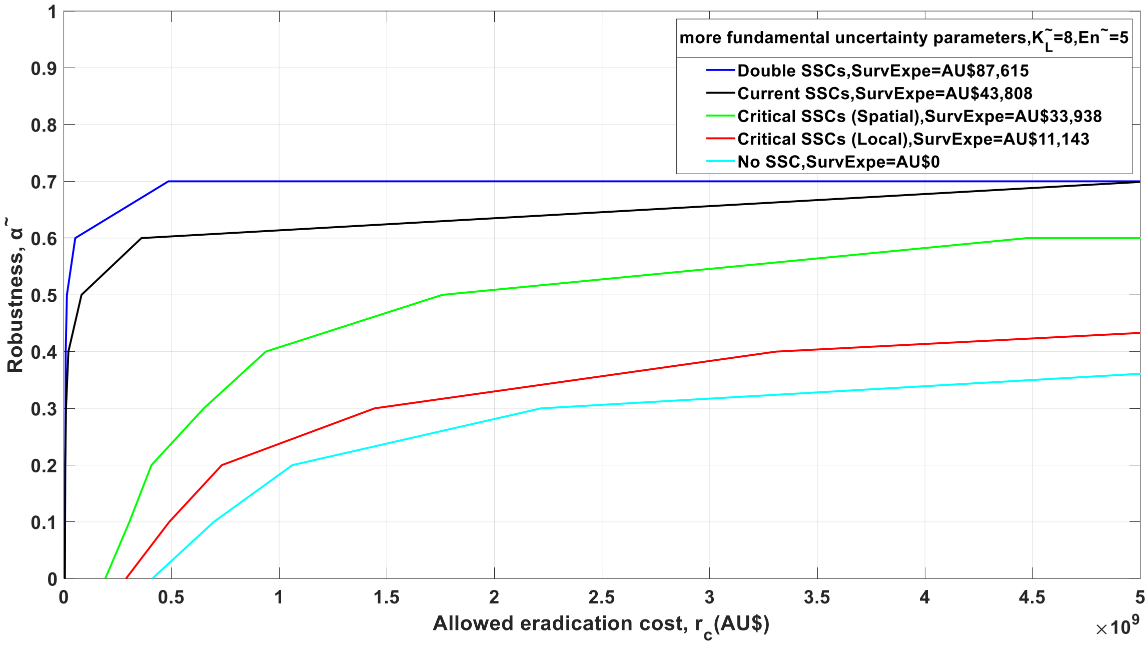

Figure S2. Robustness curves for situations shown in the legend with uncertainties modelled in more fundamental parameters. In each situation, the population threshold of Asian house gecko (AHG) at each location is estimated at 8 (i.e. $\tilde{K}_L = 8$ for $L = 1,\ldots,6$) and annual number of entries of AHG individuals after border inspection to any of the locations on Barrow Island is estimated at 5 ($\tilde{En} = 5$). All lines, except for the red line (based on local spread model), are based on spatial spread model. The optimal allocation of Surveillance System Components (SSCs) in each zone at each location is denoted as 'Optimal Spatial'. The application of double (200%) and no SSC in each zone at each location is denoted as 'Doubling Optimal Spatial' and 'No SSCs Spatial' separately. The 'Critical Spatial' and 'Critical Local' are applied to meet Chevron's ministerial requirements. The 'SurvExpe' indicates the corresponding estimated surveillance cost in each situation

Reference

1. Thomas, M. L. *et al.* Many eyes on the ground: citizen science is an effective early detection tool for biosecurity. *Biol. Invasions* **19,** 2751-2765 (2017).
